# *Mycobacterium avium* Complex Infection in a Patient with Sickle Cell Disease and Severe Iron Overload

**DOI:** 10.1155/2014/405323

**Published:** 2014-12-04

**Authors:** Kamal Shemisa, Nasima Jafferjee, David Thomas, Gretta Jacobs, Howard J. Meyerson

**Affiliations:** ^1^University of Texas Southwestern University Hospital, 5323 Harry Hines Boulevard, Dallas, TX 75390, USA; ^2^Yale University Hospital, 20 York Street, New Haven, CT 06510, USA; ^3^University Hospitals Case Western Reserve University, 11100 Euclid Avenue, Cleveland, OH 44106, USA

## Abstract

A 34-year-old female with sickle cell anemia (hemoglobin SS disease) and severe iron overload presented to our institution with the subacute presentation of recurrent pain crisis, fever of unknown origin, pancytopenia, and weight loss. A CT scan demonstrated both lung and liver nodules concerning for granulomatous disease. Subsequent biopsies of the liver and bone marrow confirmed the presence of noncaseating granulomas and blood cultures isolated *Mycobacterium avium* complex MAC. Disseminated MAC is considered an opportunistic infection typically diagnosed in the immunocompromised and rarely in immunocompetent patients. An appreciable number of mycobacterial infection cases have been reported in sickle cell disease patients without immune dysfunction. It has been reported that iron overload is known to increase the risk for mycobacterial infection in vitro and in vivo studies. While iron overload is primarily known to cause end organ dysfunction, the clinical relationship with sickle cell disease and disseminated MAC infection has not been reported. Clinical iron overload is a common condition diagnosed in the sub-Saharan African population. High dietary iron, genetic defects in iron trafficking, as well as hemoglobinopathy are believed to be the etiologies for iron overload in this region. Patients with iron overload in this region were 17-fold more likely to die from *Mycobacterium tuberculosis*. Both experimental and clinical evidence suggest a possible link to iron overload and mycobacterial infections; however larger observational studies are necessary to determine true causality.

## 1. Background

Clinicians caring for patients suffering from sickle cell disease use a disease specific management strategy when treating the illness. While treatment in the acute setting is generally supportive, the long term goals of care focus on preventing major complications of the disease. The consequences of recurrent sickling within the body's small vascular beds are hemolysis, acute chest syndrome, pulmonary hypertension, renal failure, avascular necrosis, and priapism. Greater susceptibility to infection is largely attributed to functional asplenia, hydroxyurea related immunosuppression, port related colonization from nosocomial bacteria, and malnutrition. Lastly, iron overload can result in restrictive cardiomyopathy, cirrhosis, myelosuppression, and gonadal dysfunction.

Disseminated mycobacterial infections are considered opportunistic infections typically diagnosed in the immunocompromised state and rarely in immunocompetent patients. Infections typically occur in solid organ transplant recipient patients, the critically ill, HIV/AIDS, and the very elderly [1–4]. Susceptibility to mycobacterial infection is therefore caused by dysfunctions in cell-mediated immunity. Only a modest number of mycobacterial infections have been reported in sickle cell disease. Those patients however were otherwise immunocompentent which raises the query of an alternative susceptibility in this patient population [5–8]. While sickle cell disease patients are known to be vulnerable to infections, there lacks explanation for susceptibility to mycobacterial organisms.

Patients with iron overload however were 17-fold more likely to die from* Mycobacterium tuberculosis *[9]. Interestingly, experimental models have described enhanced mycobacterial growth within iron rich environments [10–12].* Mycobacterium* can acquire iron via unique pathophysiological mechanisms which then promote survival and growth of the organism. The associations of significant iron overload with this type of infection and sickle cell disease has not been reported in the clinical literature. In order to shed light on this rather underappreciated clinical phenomenon, we present an interesting patient with sickle cell disease and severe iron overload who was subsequently diagnosed with disseminated* Mycobacterium avium *complex (MAC).

## 2. Case Presentation

A 34-year-old female with sickle cell anemia (hemoglobin SS disease) presented to our institution with recurrent pain crisis, three month (18 lbs/8.2 kg) weight loss, and fever of unknown origin (101.5 F/38.6 C). Her medical history includes secondary iron overload complicated by restrictive cardiomyopathy diagnosed on echocardiography and early stage cirrhosis diagnosed on ultrasound. Serum ferritin levels were measured over the course of one year and considered markedly elevated [13,000 mcg/L, (female normal 12–150 mcg/L)]. She was treated with oral deferasirox and hydroxyurea but only achieved minimal reductions in serum ferritin (9,454 mcg/L).

**Figure 1:**
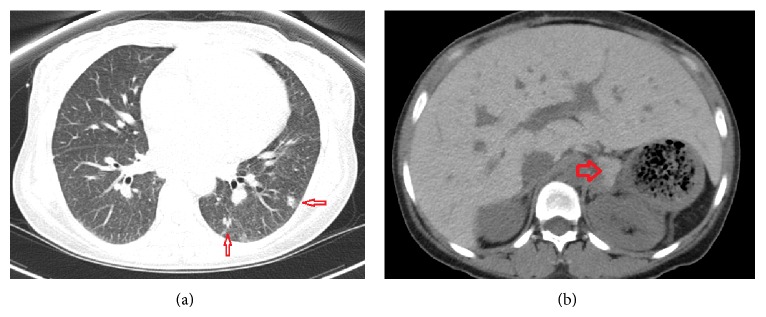
Axial noncontrast CT scan through the lung bases and upper abdomen. (a) Multiple pulmonary nodules are noted in the left lung base (small red arrows). (b) The liver and left adrenal gland (large red arrow) are hyperdense on the noncontrast exam, a finding that is consistent with iron overload.

**Figure 2:**
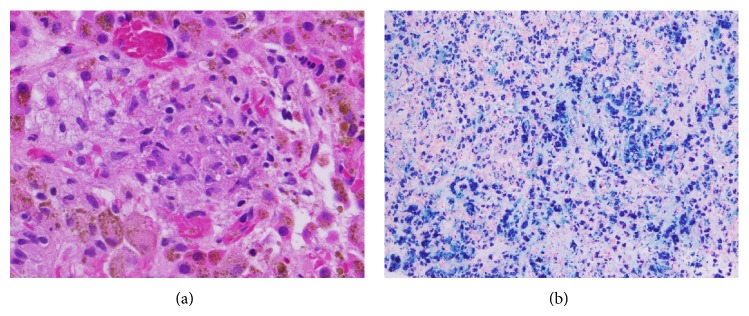
(a) Liver biopsy demonstrating large noncaseating granuloma. (b) Prussian blue staining for iron elements confirms severe hemosiderosis.

The initial investigation included computed tomography (CT) of the chest/abdomen/pelvis which was significant for an enlarged hyperdense liver and left adrenal gland. These features were consistent with hepatic hemosiderosis. (Figure 1). Other findings included multiple lung nodules as well as nonspecific axillary, mediastinal, and periportal lymphadenopathy. She then underwent an uncomplicated liver biopsy which was histopathologically consistent for severe secondary hemosiderosis with noncaseating granulomas (Figure 2). The clinical, radiographic, and pathological findings initially supported the diagnosis for sarcoidosis with underlying iron overload of which systemic corticosteroids (Prednisone 60 mg) were prescribed. An HIV test was ordered prior to initiation of therapy and was nonreactive. There was minimal clinical improvement with steroid treatment and she subsequently developed new pancytopenia.

**Figure 3:**
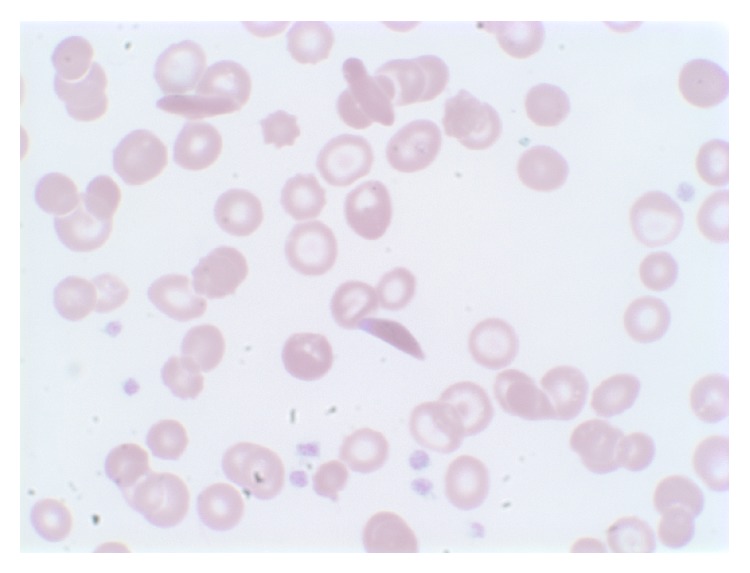
Peripheral blood smear of patient on high magnification shows irreversible sickled cells, anisocytosis, poikilocytosis, and target cells.

**Figure 4:**
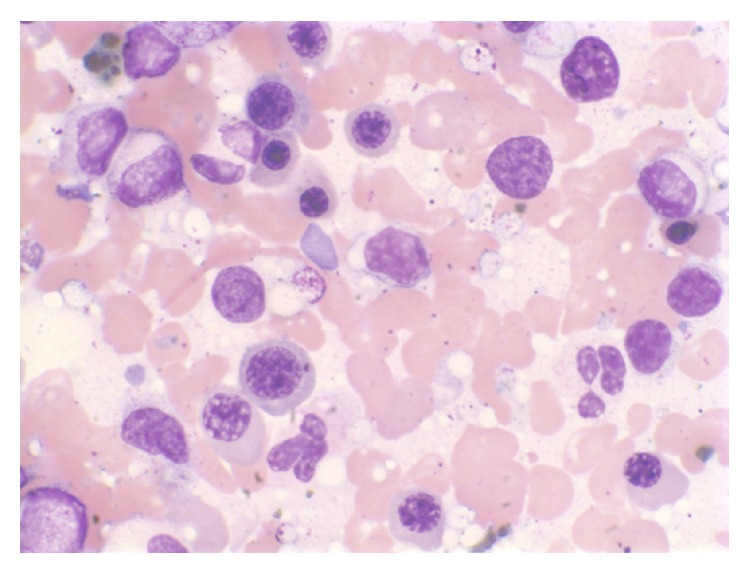
Peripheral bone marrow smear on high magnification shows erythroid hyperplasia.

**Figure 5:**
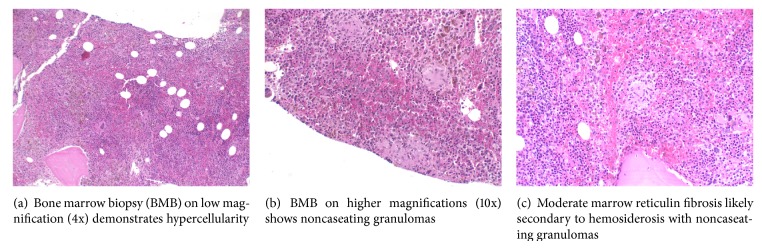
Bone marrow biopsy.

A complete hematological evaluation followed including a blood smear and bone marrow biopsy (Figures 3, 4, and 5) which was consistent with hypercellularity, severe hemosiderosis, and noncaseating granulomas. Re-treatment of sarcoidosis was initially considered based on the histological findings however blood culture results were significant for growth of acid fast bacilli. The species of bacteria was later confirmed to be* Mycobacterium avium* complex (MAC). She was subsequently treated for disseminated MAC with combination rifabutin, ethambutol, and clarithromycin. There was significant clinical improvement shortly after initiation of therapy. She was appropriately monitored and treated in our infectious disease clinic for one year.

## 3. Discussion

Disseminated MAC infection rarely occurs in immune-competent patients. Susceptibility to disseminated MAC is due to a variety of host and environmental risk factors. These risk factors include critical illness, functional asplenia, HIV infection, prolonged course of corticosteroid therapy, and anti-TNF therapy [1]. In sickle cell disease, susceptibility to mycobacterial infection may be related to iron overload. As will be described, iron overload provides a survival advantage for* Mycobacterium*. In the absence of the typical risk factors for susceptibility, one should consider iron overload as a contributor to disseminated MAC infection in this patient. 

*Iron Trafficking, the Host Defense, and Mycobacterium Pathogenicity. *The role iron trafficking plays in our understanding of the immune system is relatively underappreciated. Iron cations are required to perform essential functions within microorganisms. Limiting the access of microorganisms to iron is an evolutionary strategy of the host defense. Iron utilization and trafficking involve the chelation of extracellular iron by host proteins. Proteins that bind iron including transferrin (TF) and lactoferrin, are involved in the storage of intracellular iron.

Within the host, the* Mycobacterium* are phagocytized and contained within the macrophage phagosome, an iron poor environment [10]. Host mechanisms to limit iron acquisition by microorganisms involve the interaction of iron binding proteins, TfR (transferrin receptor), beta-2 microglobulin, and HFE (Class I major histocompatibility complex). The deficiencies in HFE and beta-2 microglobulin are known to raise the susceptibility to* Mycobacterium* infection [12]. In response to infection, macrophages also increase expression of natural resistance associated proteins (NRAP) [13, 14]. These cellular mechanisms again are aimed to sequester host iron and deprive bacteria dependent on iron.

Although impairing the utilization of iron via sequestration and efflux prevents the growth of bacteria, there remain pathogenic mechanisms of acquiring iron.* Mycobacterium* remove iron from host binding proteins with siderophores. These high affinity molecules remove iron from the host cell [15–17]. The release of hepcidin like molecules further augment iron acquisition via the down-regulation of ferroportin, a transport protein necessary for iron efflux from the cell [13]. Other adaptive functions include interference in host cell gene expression of Nramp, TfR, and HFE mRNA [14]. Surpluses of iron in vitro further enhance the growth of* Mycobacterium* and are known to worsen disease in people [18].

In response, infected macrophages release interferon gamma which downregulate TfR and HFE genes thus reducing the body's absorption of iron. Furthermore, macrophages synthesize siderocalin, a protein designed to inhibit the action of siderophores. Treatment of infected macrophages with siderocalin limits the replication of mycobacteria within the cell. Investigating the potential role of siderophore inhibitors in treating MAC infection may provide insight towards developing alternative therapeutic strategies [19]. While the effect of iron chelation has yet to be studied, correction of iron overload in experimental models reduces the susceptibility to* Mycobacterium* infection as compared to controls [20]. 

*Clinical Correlations of Iron Overload and Mycobacterial Infection.* Iron overload is a common condition in the sub-Saharan African population. High dietary iron and genetic defects in iron trafficking proteins as well as hemoglobinopathy are believed to be the etiologies for iron overload in this region. Patients with iron overload were 17-fold more likely to die from* Mycobacterium tuberculosis* [9]. This clinical correlate also applies to infections from other* Mycobacterium* species. In Western Africa,* Mycobacterium ulcerans* is a prevalent cause for nontuberculous skin nodules and osteomyelitis. Higher prevalence rates were found in patients in this region with sickle cell disease [6]. Finally, an outbreak study of confirmed* Mycobacterium mucogenicum* bloodstream infections indicated that the four infected sickle cell disease patients had iron overload [5].

## 4. Conclusions

Disseminated MAC infections are considered opportunistic infections typically diagnosed in the immunocompromised state. Sickle cell disease patients reported to have mycobacterial infections often do not have risk factors for susceptibility. We described to you a sickle cell disease patient with severe iron overload who was diagnosed with disseminated MAC. In experimental models, iron overload is known to overwhelm protective mechanisms for sequestration and efflux of iron thus predisposing to infection. Correction of iron overload reverses this susceptibility as compared to controls. Clinical evidence suggests a possible link to iron overload and mycobacterial infections; however larger observational studies are necessary to determine true causality. Future experimental/clinical endeavors can then be geared towards discovering therapeutic drugs which target mechanisms of iron-acquisition by the bacteria.
